# Case Report: Arterial Wall Inflammation in Atherosclerotic Cardiovascular Disease is Reduced by Olamkicept (sgp130Fc)

**DOI:** 10.3389/fphar.2022.758233

**Published:** 2022-06-09

**Authors:** Dominik M. Schulte, Georg H. Waetzig, Harald Schuett, Marlies Marx, Berenice Schulte, Christoph Garbers, Juliane Lokau, Ann-Kathrin Vlacil, Juliane Schulz, Anna K. Seoudy, Bernhard Schieffer, Philip Rosenstiel, Marcus Seeger, Matthias Laudes, Stefan Rose-John, Ulf Lützen, Karsten Grote, Stefan Schreiber

**Affiliations:** ^1^ Department of Internal Medicine I, University Medical Center Schleswig-Holstein (UKSH), Kiel, Germany; ^2^ Institute of Diabetes and Clinical Metabolic Research, Kiel University and UKSH, Kiel, Germany; ^3^ Institute of Clinical Molecular Biology, Kiel University and UKSH, Kiel, Germany; ^4^ CONARIS Research Institute AG, Kiel, Germany; ^5^ Department of Cardiology and Angiology, Philipps-University, Marburg, Germany; ^6^ Department of Nuclear Medicine, Molecular Imaging Diagnostics and Therapy, UKSH, Kiel, Germany; ^7^ Department of Pathology, Otto-von-Guericke-University, Magdeburg, Germany; ^8^ Biochemical Institute, Kiel University, Kiel, Germany

**Keywords:** atherosclerosis, case report, interleukin-6, olamkicept, sgp130Fc

## Abstract

Inflammation is a strong driver of atherosclerotic cardiovascular disease (ASCVD). There is a large unmet need for therapies that prevent or reduce excessive inflammation while avoiding systemic immunosuppression. We showed previously that selective inhibition of pro-inflammatory interleukin-6 (IL-6) trans-signalling by the fusion protein olamkicept (sgp130Fc) prevented and reduced experimental murine atherosclerosis in low-density lipoprotein receptor-deficient (*Ldlr* ^−/−^) mice on a high-fat, high-cholesterol diet independently of low-density lipoprotein (LDL) cholesterol metabolism. Therefore, we allowed compassionate use of olamkicept (600 mg intravenously biweekly for 10 weeks) in a patient with very-high-risk ASCVD. Despite optimal LDL cholesterol under maximum tolerated lipid-lowering treatment, the patient had a remaining very high risk for future cardiovascular events related to significant arterial wall inflammation with lipoprotein (a) [Lp(a)]-cholesterol as the main contributor. ^18^Fluorodeoxyglucose positron emission tomography/computed tomography (^18^FDG PET/CT) measurements were performed before and after the treatment period. Olamkicept reduced arterial wall inflammation in this patient without interfering with lipoprotein metabolism. No clinical or laboratory side effects were observed during or after treatment with olamkicept. Our findings in this patient matched the results from our mechanistic study in *Ldlr* ^−/−^ mice, which were extended by additional analyses on vascular inflammation. Olamkicept may be a promising option for treating ASCVD independently of LDL cholesterol metabolism. A Phase II trial of olamkicept in ASCVD is currently being prepared.

## Introduction

Anti-cytokine therapy is a promising option for treating ASCVD that is progressive despite lifestyle modification and optimizing plasma lipid levels (Schuett and Schieffer 2012; Ait-Oufella et al., 2019; Soehnlein and Libby 2021). The challenge in ASCVD is to diminish local, LDL cholesterol-driven, self-perpetuating metabolic inflammation in atherosclerotic plaques and, at the same time, to preserve systemic immune defense. The CANTOS trial investigated the anti-interleukin-1β (IL-1β) antibody canakinumab in established human inflammatory ASCVD and demonstrated the challenge of significant benefits through lowering the rate of recurrent cardiovascular events at the expense of a higher incidence of fatal infections (Ridker et al., 2017). Downstream of IL-1β, IL-6 signalling is centrally involved in atherogenesis (Libby 2017). IL-6 exerts its multiple functions through two main signalling pathways, which both require signal transduction by the transmembrane co-receptor gp130 (Scheller et al., 2014). In classic signalling, IL-6 uses the membrane-bound IL-6 receptor (IL-6R), which is mainly expressed by hepatocytes and some leukocyte subsets. In the trans-signalling pathway, circulating soluble IL-6R (sIL-6R) recruits IL-6 to form IL-6/sIL-6R complexes, which could activate the ubiquitously expressed gp130 on nearly any cell in the body (Garbers et al., 2018). However, such ubiquitous trans-signalling is physiologically prevented by an excess of soluble gp130 isoforms (sgp130) acting as a buffer in the blood (Zhou et al., 2020). While classic IL-6 signalling has many physiological and anti-infectious functions, excessive trans-signalling is seen in many chronic inflammatory conditions (Rose-John et al., 2017; Garbers et al., 2018). Specific trans-signalling inhibition instead of blocking IL-6 or its receptor has therefore been proposed to treat chronic inflammation without the negative effect of systemic immunosuppression (Rose-John et al., 2017; Garbers et al., 2018).

We developed the selective IL-6/sIL-6R complex trap sgp130Fc consisting of two sgp130 domains fused to the crystallisable fragment of human immunoglobulin G1 (Jostock et al., 2001). Olamkicept (optimized sgp130Fc) was efficacious in a large series of animal models (Rose-John et al., 2017; Garbers et al., 2018) and successfully passed Phase I trials without safety issues (EudraCT no.s 2012-005142-38 and 2013-004208-20). In a recently published Phase IIa trial in patients with inflammatory bowel diseases, the mechanism of action of olamkicept known from animal models of colitis was confirmed and olamkicept demonstrated clinical efficacy (Schreiber et al., 2021), which was recently confirmed in a larger Phase IIb trial in ulcerative colitis (NCT03235752). Patients with ASCVD show a dysregulated IL-6/sIL-6R/sgp130 system with decreased sgp130 levels (Schuett et al., 2012). Olamkicept specifically blocks IL-6 trans-signalling and expands the serum sgp130 buffer (Rose-John et al., 2017; Garbers et al., 2018). Our previous investigations in a standard murine atherosclerosis model (LDL receptor-deficient [*Ldlr* ^*−/−*^] mice on a high-fat, high-cholesterol diet) showed both preventive and therapeutic efficacy of olamkicept, notably with a significant regression of established atherosclerotic lesion size (Schuett et al., 2012). Importantly, it has recently been shown in a rat myocardial infarction (MI) model that rat sgp130Fc but not an anti-IL-6-antibody attenuated neutrophil and macrophage infiltration into the myocardium, reduced infarct size, and preserved cardiac function 28 days after MI (George et al., 2021).

These data in rodents could lead to closing a therapeutic gap in humans. About 50% of ASCVD patients are left with an untreated residual inflammatory risk, a crucial part of the ASCVD treatment paradigm (Ridker et al., 2017), and atherosclerotic plaque burden. Thus, clinical cases with high inflammatory ASCVD and plaque burden remain untreated with licensed anti-ASCVD medical treatment. Crucial mediators of arterial wall inflammation are proinflammatory oxidized phospholipids carried by Lp(a) (van der Valk et al., 2016). Therefore, Lp(a) is an independent and causal risk factor for ASCVD (Tsimikas 2017; Stiekema et al., 2019), related to cardiovascular death, myocardial infarction, and stroke when high-sensitivity C-reactive protein (hsCRP) levels are 2 mg/L or more (Puri et al., 2020). Today, there is no medical option to treat Lp(a) and hsCRP in ASCVD patients (Tsimikas and Stroes 2020). Although anti-proprotein convertase subtilisin/kexin type 9 (PCSK9) therapy can reduce Lp(a) by about 20–30%, the anti-PCSK9 antibody evolocumab did not influence arterial wall inflammation (Stiekema et al., 2019). Only Lp(a) apheresis seemed to lower Lp(a) and hsCRP levels, resulting in reduction of the atherosclerotic burden (Pokrovsky et al., 2017).

## Case Description

**FIGURE 1:**
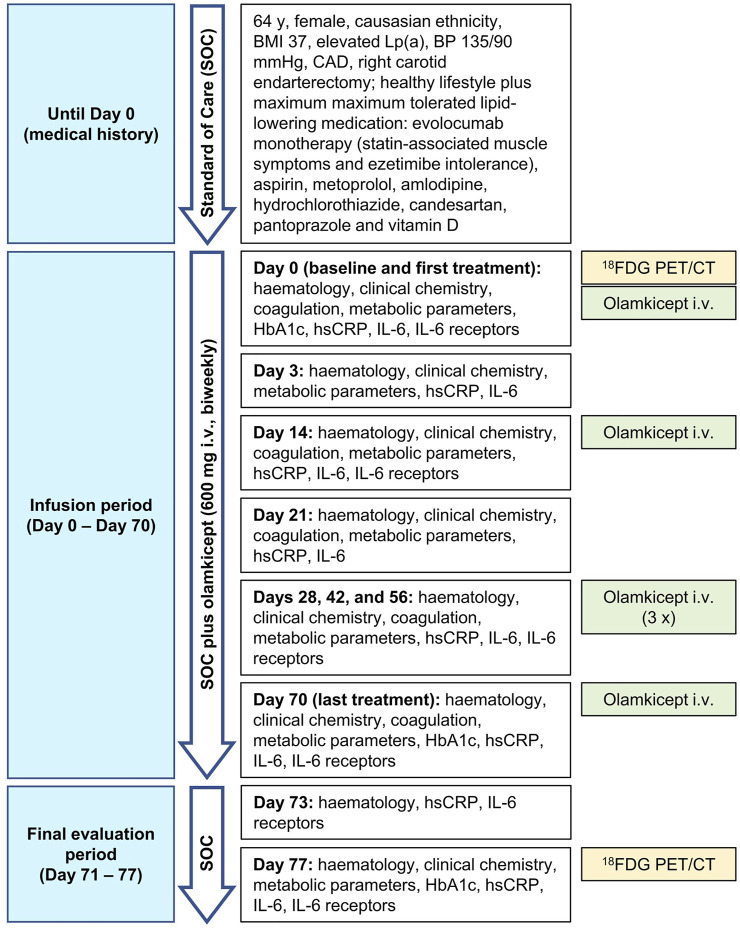
Case report timeline. BMI, body mass index; BP, blood pressure; CAD, coronary artery disease; ^18^FDG PET/CT, ^18^Fluorodeoxyglucose positron emission tomography/computed tomography; HbA1c, glycated hemoglobin; hsCRP, high-sensitivity C-reactive protein; IL-6, interleukin-6; i.v., intravenously.

Due to the large unmet need for adding anti-cytokine treatments to lipid reduction therapies for ASCVD (Ridker 2017), we allowed compassionate use of olamkicept (600 mg intravenously biweekly for 10 weeks) in a patient with very-high-risk ASCVD (Mach et al., 2020), elevated Lp(a) and high inflammatory vascular risk (Ridker 2016) under maximum tolerated lipid-lowering treatment. The patient was a Caucasian female aged 64 years (body mass index: 37 kg/m^2^, blood pressure 135/90 mmHg), with very-high-risk ASCVD (ANA/ANCA-negative). She had a history of coronary artery disease and had previously undergone right carotid endarterectomy. The patient’s therapy consisted of evolocumab, aspirin, metoprolol, amlodipine, hydrochlorothiazide, candesartan, pantoprazole, vitamin D and a healthy lifestyle. Type 2 diabetes mellitus was controlled without medication as a choice of the patient at this stage. Non-alcoholic fatty liver disease (NAFLD) seemed unlikely in this patient due to low alanine aminotransferase (Ma et al., 2020) and non-detectable IL-11 serum levels (Dong et al., 2021) (Table 1) as well as the absence of any signs of NAFLD in the low dose CT. Despite maximum tolerated lipid-lowering treatment (evolocumab monotherapy because of statin-associated muscle symptoms and ezetimibe intolerance), the patient still had a remaining very high risk for future vascular events related to the advanced stage of ASCVD because of elevated hsCRP and Lp(a). The patient’s characteristics are detailed in Table 1. Olamkicept was administered open-label at the clinical trial dose—600 mg intravenously (i.v.) within 1 h, biweekly (Schreiber et al., 2021)—for 10 weeks (6 infusions) from January to March 2018 (Table 1; Figure 1). Olamkicept’s half-life is 4.7 days. The patient was monitored for infusion reactions for 3 h (first two infusions) or 1 h (subsequent infusions).

**TABLE 1:** Patient’s characteristics, treatment and diagnostics.

Days	0	3	14	21	28	42	56	70	73	77
Olamkicept infusion (600 mg)	x	—	x	—	x	x	x	x	—	—
^18^Fluorodeoxyglucose positron emission tomography/computed tomography—whole body	x	—	—	—	—	—	—	—	—	x
Leukocytes [x10^9^/L]	10.7	10.4	10.2	10.8	11.0	10.7	11.9	10.3	8.54	11.0
Immature granulocytes [x10^9^/L]	0.03	0.05	0.03	0.04	0.04	0.05	0.06	0.04	0.04	0.05
Neutrophils [x10^9^/L]	7.3	6.9	7.1	7.3	7.7	7.6	8.2	7.2	5.4	7.8
Eosinophils [x10^9^/L]	0.2	0.4	0.2	0.3	0.2	0.2	0.2	0.1	0.2	0.2
Basophils [x10^9^/L]	0.1	0.1	0.1	0.1	0.1	0.1	0.1	0.1	0.1	0.1
Lymphocytes [x10^9^/L]	2.5	2.5	2.3	2.5	2.4	2.3	3.7	2.3	2.5	2.2
Monocytes [x10^9^/L]	0.6	0.5	0.6	0.6	0.6	0.6	0.6	0.6	0.4	0.6
Haemoglobin [g/dL]	15.6	16.7	15.7	16.1	15.7	15.3	15.5	15.9	15.6	16.1
Platelets [x10^9^/L]	200	203	173	179	193	175	178	195	84	191
Sodium [mmol/L]	141	140	143	143	139	140	144	140	—	—
Potassium [mmol/L]	3.84	3.75	3.91	3.99	4.05	3.94	4.03	3.92	—	—
Calcium, albumin-corrected [mmol/L]	2.38	2.44	2.44	—	—	—	2.54	2.49	—	2.4
Glomerular filtration rate [mL/min/1.73]	60	68	60	67	73	63	60	63	—	—
Apolipoprotein B [mg/dL]	51	–	56.0	61.0	53.0	51.0	51.0	53.0	—	66.0
Bilirubin [µmol/L]	7.4	8.3	9.1	9.0	7.4	8.5	5.1	8.8	—	—
Creatine kinase [U/L]	42	37	28	37	30	27	54	35	—	—
Alanine aminotransferase [U/L]	13.8	15.5	15.0	16.8	13.0	19.0	15.9	13.8	—	—
γ-glutamyl transferase [U/L]	40	41	32	39	39	37	38	40	—	—
Lipase [U/L]	33	41	33	34	36	32	29	33	—	—
International Normalized Ratio	1.01	—	1.02	0.99	0.96	1.05	1.01	1.06	—	—
D-dimer [mg/L]	0.34	—	0.31	0.34	0.47	0.32	0.26	0.32	—	—
Cholesterol [mmol/L]	2.7	3.1	2.9	3.1	2.9	2.6	2.6	2.7	—	3.0
HDL cholesterol [mmol/L]	1.0	1.15	1.08	1.05	1.03	0.97	1.04	0.97	—	1.19
LDL cholesterol [mmol/L]	1.3	1.65	1.4	1.62	1.36	1.21	1.27	1.34	—	1.48
Triglycerides [mmol/L]	2.2	2.2	2.0	2.4	2.0	2.2	2.1	2.2	—	1.7
Lipoprotein (a) [nmol/L]	238.7	250.2	227.2	236.7	241.1	234.2	225.7	243.0	—	231.7
hsCRP (high-sensitivity C-reactive protein) [mg/dL]	9.18	3.33	12.7	7.06	9.99	11.2	18.9	14.4	4.1	7.14
Interleukin-6 [pg/mL]	4.8	32.0	9.8	24.3	12.0	11.7	15.5	11.7	40.6	26.8
Soluble interleukin-6 receptor [ng/mL]	60.84	—	61.18	—	66.67	70.45	53.86	60.84	64.15	54.54
Soluble gp130 (including olamkicept) [ng/mL]	319.12	—	983.41	—	1,094.79	1,281.33	1,061.24	1,014.27	2,620.65	1865.10
IL-11 [pg/ml]	< LOD	—	< LOD	—	< LOD	< LOD	< LOD	< LOD	< LOD	< LOD
HbA1c (glycated hemoglobin) [%]	6.7	—	—	—	—	—	—	—	—	6.5

<LOD, below the limit of detection.

For clinical assessment and non-invasive imaging, we used ^18^FDG PET/CT. In the patient, screening for inflammatory ASCVD consisted of a whole body ^18^FDG PET/CT examination. ^18^FDG PET/CT has shown great potential in visualizing, quantifying and characterizing atherosclerotic inflammation and plaque stability non-invasively, emerging as a suitable surrogate endpoint for clinical testing of novel anti-atherosclerotic therapeutics (Rudd et al., 2007; Rudd et al., 2008; Moreno Velasquez et al., 2015; Ali and Tawakol 2016; Hyafil and Vigne 2019). For example, arterial wall inflammation measured by ^18^FDG PET/CT decreased by testing statins (Tawakol et al., 2013), lipoprotein apheresis (van Wijk et al., 2014) and IL-1β inhibition (Hsue et al., 2018), whereas arterial wall inflammation persisted under anti-PCSK9 antibody treatment (Stiekema et al., 2019) and increased under glucocorticoid therapy (van der Valk et al., 2015). The target-to-background ratio (TBR) was calculated as described previously (van Wijk et al., 2014).

In this patient, 600 mg olamkicept administered i. v. biweekly over 10 weeks was safe. No clinical or laboratory side effects were observed during or after treatment (Table 1). Also from the patient’s perspective, the infusions were well tolerated. While sIL-6R levels remained unchanged, concentrations of serum IL-6 increased slightly, reflecting olamkicept’s additional sgp130 buffering capacity for IL-6/sIL-6R complexes (Table 1). Administration of olamkicept transiently decreased elevated high-sensitivity C-reactive protein (hsCRP) by 64% 3 days after infusion (Table 1). A missing long-lasting effect on hsCRP suppression may be explained by the pharmacokinetics of olamkicept and by the fact that CRP production is controlled by classic IL-6 signalling, which is not inhibited by olamkicept (Hoge et al., 2013; Garbers et al., 2018). Therefore, the reduction in hsCRP observed after olamkicept treatment does not represent a mechanistic reduction by blocking IL-6-mediated CRP production, but rather reflects a reduced inflammatory burden. As expected for selective inhibition of IL-6 trans-signalling, serum levels of total cholesterol, high-density lipoprotein (HDL) cholesterol, LDL cholesterol, triglycerides and Lp(a) did not show any clear trends or changes under olamkicept treatment (Table 1). This is in contrast to the common anabolic side effects (increased serum triglyceride and cholesterol levels as well as body weight) observed with anti-IL-6 or anti-IL-6R, which inhibit both classic and trans-signalling (Garbers et al., 2018).

**FIGURE 2:**
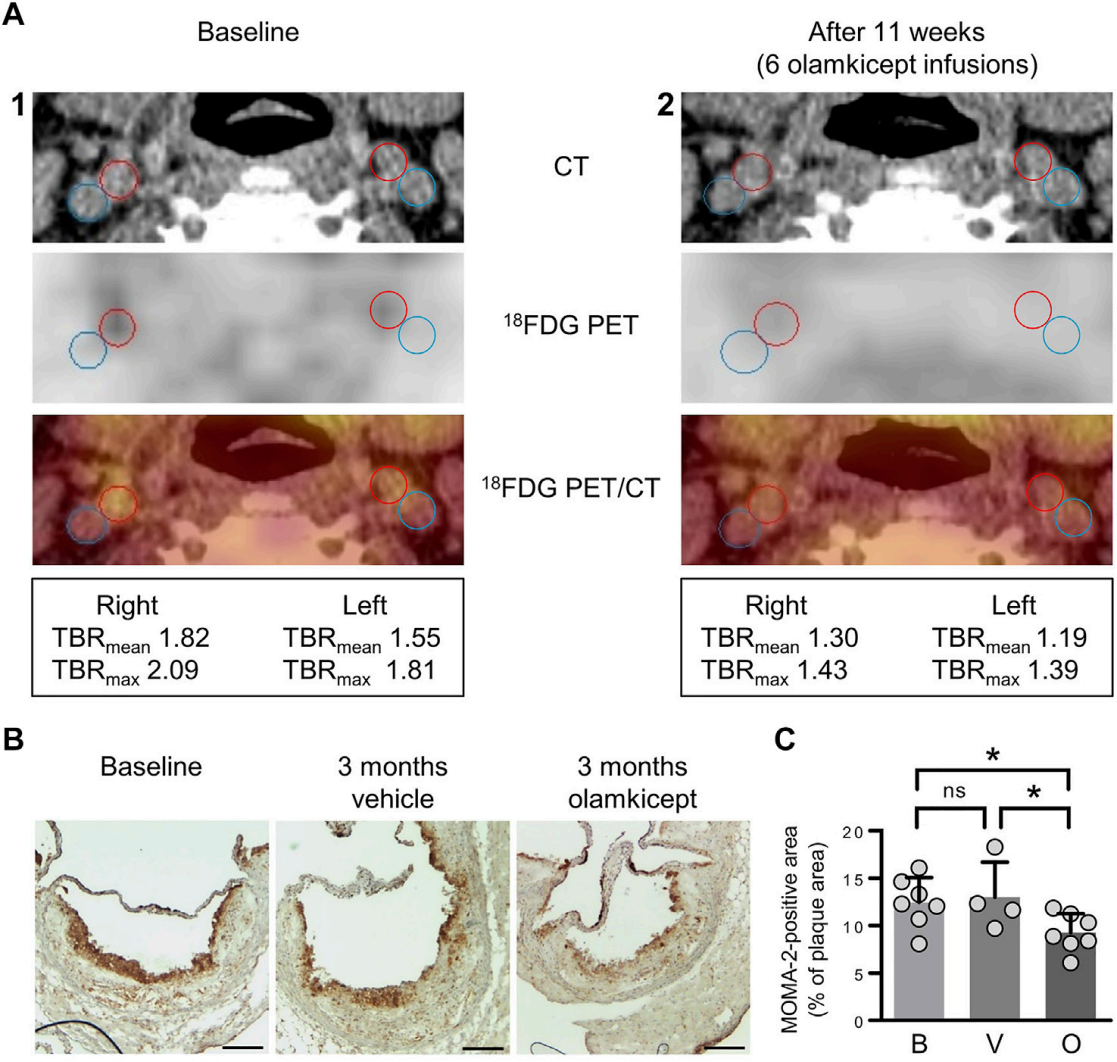
Inhibition of IL-6 trans-signalling reduces arterial wall inflammation and macrophage infiltration of atherosclerotic plaques in end-stage atherosclerosis. **(A)** Arterial wall inflammation in the carotid arteries of the patient (1) at baseline and (2) 11 weeks after the beginning of olamkicept treatment (6 infusions of 600 mg i. v. biweekly; Table 1). In the representative axial computed tomography (CT), ^18^fluorodeoxyglucose positron emission tomography (^18^FDG PET), and fused images (^18^FDG PET/CT), regions of interest are highlighted by red (artery) and blue circles (vein). Mean and maximum target-to-background ratio (TBR_mean_ and TBR_max_) are listed below. **(B,C)** New analyses of samples from a previous study in a murine model of experimental atherosclerosis (Schuett et al., 2012) **(B)** Representative MOMA-2-stained (macrophage-positive) lesions in aortic root sections (scale bars = 250 µm) of *Ldlr* ^−/−^ mice (*n* = 4 to *n* = 7 per group) after 12 weeks on a high-fat, high-cholesterol diet followed by another 12 weeks on the same diet combined with vehicle or olamkicept treatment (0.5 mg/kg intraperitoneally, twice weekly); **(C)** MOMA-2 levels expressed as percentage (mean, standard deviation) of the MOMA-positive (+) total plaque area at baseline (B) or after vehicle (V) or olamkicept treatment (O); *, *p* < 0.05; ns, not significant.

We had previously shown that olamkicept reduced cellular infiltration of plaques by cells positive for the monocyte/macrophage marker MOMA-2 (mainly macrophages) early in the course of experimental atherosclerosis in mice (Schuett et al., 2012). Therefore, we compared ^18^FDG PET/CT images of arterial wall inflammation in the carotid arteries in this patient before and after compassionate use of olamkicept (6 biweekly infusions; Table 1; Figure 1). The density of plaque macrophages has been shown to correlate with the uptake of ^18^FDG measured by PET (Moreno Velasquez et al., 2015), and the resulting signal is expressed as mean and maximum target-to-background ratio (TBR_mean_ and TBR_max_). The patient presented with an atherosclerosis characterized by Lp(a)-cholesterol and hsCRP. The arterial wall inflammation detected by ^18^FDG PET/CT at baseline was strongly reduced after 3 months by 6 infusions of olamkicept (Figure 2A) without observing changes in lipoprotein distribution. This is in contrast to evolocumab treatment, where a large reduction in LDL cholesterol and a small reduction in Lp(a) levels did not lead to a decrease in arterial wall inflammation (Stiekema et al., 2019). Similar to the ^18^FDG PET/CT findings in this patient, olamkicept decreased the amount of MOMA-2-positive plaque macrophages in atherosclerotic lesions also in samples from mice with established experimental atherosclerosis which had been produced in our previous mechanistic trial (Schuett et al., 2012) but not yet been analysed in this regard (Figures 2B,C).

Taken together, the specific therapeutic inhibition of IL-6 trans-signalling in established atherosclerosis reduced local inflammatory activity and thus the atherosclerotic burden in a patient with very-high-risk ASCVD despite maximum tolerated lipid-lowering medical treatment. The patient has been regularly visiting our outpatient clinic until today (last visit in week 16, 2022). So far, no clinical progression of the very-high-risk ASCVD or any cardiovascular events occurred.

## Discussion

ASCVD is primarily caused by an interplay of lipid metabolism disorder and inflammatory atherosclerosis (Ross 1999). Patients with very-high-risk ASCVD and high inflammatory load despite state-of-the-art medical treatment have a large unmet need for effective anti-inflammatory therapies. Specific inhibition of IL-1β can be beneficial in patients with inflammatory atherosclerosis (Ridker et al., 2017), in contrast to general immunosuppression (*e.g.*, by methotrexate) (Ridker et al., 2019), which does not reduce cardiovascular events.

IL-6 is a pleiotropic cytokine produced in response to infection and tissue damage. Patients with ASCVD show increased levels of circulating IL-6, which are correlated with clinical activity (Ridker 2016). High IL-6 plasma levels are associated with a higher risk of future cardiovascular events (Kaptoge et al., 2014). In murine models, lifetime IL-6 deficiency was atheroprotective (Madan et al., 2008) and inhibition of IL-6R reduced atherosclerotic lesions (Akita et al., 2017). Selective IL-6 trans-signalling inhibition, but not global IL-6 blockade, is efficacious in ameliorating the consequences of MI in a rat model and preserving cardiac function (George et al., 2021). In human atherosclerosis, inhibition of IL-1β by canakinumab led to a significantly lower rate of recurrent cardiovascular events and lowered IL-6 levels. However, side effects due to the systemic immunosuppression by canakinumab led to an unfavourable risk/benefit ratio for the therapy of ASCVD (Ridker et al., 2017; Palmer and Vaccarezza 2019). These results are in line with the increased rate of opportunistic and severe infections that is observed with the anti-IL-6R antibody tocilizumab (Rose-John et al., 2017). Another potential limitation of complete IL-6 inhibition is the potential increase in triglycerides and LDL cholesterol (Garbers et al., 2018; Soehnlein and Libby 2021).

We have previously shown in experimental atherosclerosis in *Ldlr* ^–/–^ mice that inhibiting IL-6 trans-signalling by olamkicept can both prevent atherogenesis and reduce established atherosclerosis, which is a rare finding in a disease model (Schuett et al., 2012). Of note, we have now used samples from this study showing that olamkicept reduced plaque macrophage contents in a setting of established atherosclerosis. In our patient, histological validation of atherosclerotic plaque morphology was not available. In future clinical trials, coronary burden and atherosclerotic plaque characteristics shall be investigated by appropriate imaging techniques. Therefore, the planned placebo-controlled, randomized, double-blinded Phase II trial shall investigate not only the effects of olamkicept on inflammatory burden in an index vessel (carotid arteries or aorta, evaluated by ^18^FDG PET/CT), but also changes in intima-media thickness (IMT), plaque size and calcification in the carotid arteries or aorta (evaluated by 18-MHz ultrasound) as well as changes in the endothelial layer, IMT, plaque size, calcification and necrotic core in radial arteries (evaluated by 70-MHz high-resolution ultrasound).

Ziegler et al. recently demonstrated that the ratio between proinflammatory IL-6/sIL-6R and neutralized IL-6/sIL-6R/sgp130 complexes was associated with the risk of cardiovascular events, indicating that a deficit in trans-signalling buffer capacity may contribute to disease risk (Ziegler et al., 2019). This is in line with our findings that sgp130 levels are reduced in patients with coronary artery disease (Schuett et al., 2012). The slightly elevated IL-6 levels observed in our present patient mainly represent buffered IL-6 in IL-6/sIL-6R/olamkicept complexes (Garbers et al., 2018). Nevertheless, the anti-cytokine treatment olamkicept reduced arterial wall inflammation burden. It may be speculated whether a high hsCRP level would be a necessary biomarker for patient selection for a clinical trial of olamkicept in ASCVD or whether the proteins involved in IL-6 trans-signalling would be more suitable in this regard. In the planned Phase II trial, patients with CRP levels above 2 mg/dl shall be included.

The specificity and efficacy of olamkicept as a trans-signalling inhibitor was underlined by the absence of changes in general lipid profiling, especially of Lp(a) (Table 1). The data at hand from clinical trials (see Introduction) suggest to expect minimal side effects of olamkicept also in patients with very-high-risk ASCVD. In addition to high-dose i.v. induction treatment periods to quickly reduce cardiovascular inflammation and reduce plaques, also before planned cardiovascular interventions, this would also warrant permanent maintenance treatment scenarios—possibly with low-dose subcutaneous applications or more widely spaced high-dose i.v. administrations. As olamkicept does not directly inhibit the induction of acute phase proteins like CRP (Hoge et al., 2013), the decrease of hsCRP in the patient might even reflect the reduction of the systemic inflammatory state including the amelioration of inflammatory disease activity in the atherosclerotic lesions. Therefore, instead of moving upstream from IL-1β and IL-6 to identify novel targets for atheroprotection (Ridker 2016), future clinical studies should investigate specific inhibition of IL-6 trans-signalling by olamkicept. As many animal models (Garbers et al., 2018) and current human clinical data suggest that inhibition of trans-signalling does not lead to systemic immunosuppression, olamkicept might deliver a more attractive risk/benefit ratio than canakinumab for a cardiovascular benefit by directly targeting the inflammatory component of atherosclerosis independently of lipid profiles.

## Data Availability

The original contributions presented in the study are included in the article, further inquiries can be directed to the corresponding author.
